# Professional Service Utilisation among Patients with Severe Mental Disorders

**DOI:** 10.1186/1472-6963-10-141

**Published:** 2010-05-27

**Authors:** Marie-Josée Fleury, Guy Grenier, Jean-Marie Bamvita, Jean Caron

**Affiliations:** 1 Department of Psychiatry, McGill University, Researcher, Douglas Hospital Research Centre, 6875 LaSalle Blvd., Montreal, Quebec, H4H 1R3, Canada; 2 Douglas Hospital Research Centre, Montreal, Quebec, H4H 1R3, Canada

## Abstract

**Background:**

Generally, patients with serious mental disorders (SMD) are frequent users of services who generate high care-related costs. Current reforms aim to increase service integration and primary care for improved patient care and health-care efficiency. This article identifies and compares variables associated with the use by patients with SMD of services offered by psychiatrists, case managers, and general practitioners (GPs). It also compares frequent and infrequent service use.

**Method:**

One hundred forty patients with SMD from five regions in Quebec, Canada, were interviewed on their use of services in the previous year. Patients were also required to complete a questionnaire on needs-assessment. In addition, data were collected from clinical records. Descriptive, bivariate, and multivariate analyses were conducted.

**Results:**

Most patients used services from psychiatrists and case managers, but no more than half consulted GPs. Most patients were followed at least by two professionals, chiefly psychiatrists and case managers. Care access, continuity of care, and total help received were the most important variables associated with the different types of professional consultation. These variables were also associated with frequent use of professional service, as compared with infrequent service use. In all, enabling factors rather than need factors were the core predictors of frequency of service utilisation by patients with SMD.

**Conclusion:**

This study reveals that health care system organisation and professional practice - rather than patient need profiles - are the core predictors of professional consultation by patients with SMD. The homogeneity of our study population, i.e. mainly users with schizophrenia, recently discharged from hospital, may partly account for these results. Our findings also underscored the limited involvement of GPs in this patient population's care. As comorbidity is often associated with serious mental disorders, closer follow-up by GPs is needed. Globally, more effort should be directed at increasing shared-care initiatives, which would enhance coordination among psychiatrists, GPs, and psychosocial teams (including case managers). Finally, there is a need to increase awareness among health care providers, especially GPs, of the level of care required by patients with disabling and serious mental disorders.

## Background

The prevalence of mental disorders worldwide ranges from 4.3% to 26.4% per year; these conditions represent 12% of the disease burden [1]. Severe mental disorders (SMD, e.g. schizophrenia) are less prevalent (2-3% of the population), but they account for close to half of total mental health care costs [2]. The burden of SMD has prompted countries to improve their mental health care system by strengthening community-based services and primary care for these patients. Essentially, these reforms encourage general practitioners (GPs) and case managers (usually a nurse or social worker) to follow more patients with SMD in the community [1,3]. Primary care and community-based services are considered less stigmatising, more accessible, and no costlier than hospital-based care; in addition, they are often more greatly appreciated by patients [4-6]. Yet, hospitals and psychiatric care continue to occupy a central place in the mental health care system [7,8]. Most patients with SMD are still treated by psychiatrists [9]. However, most patients who live in the community are followed by case managers whose main function is to reduce hospital admission, increase use of community-based services, and enhance their patients' quality of life [10]. Finally, as chronic health problems are closely associated with SMD, a large proportion of these patients receive physical care as well as mental health aftercare from a GP [11]. In the United Kingdom, where primary care and specialised mental health services are closely integrated [12], up to 40% of patients with SMD rely mainly on a GP for their medical care and psychiatric medication [11,13]. Generally, however, patients with SMD, who are burdened with chronic disease and major functional disability, need substantial help from various resources on a long-term basis to meet their multiple bio-psycho-social needs [14].

Patterns of mental health care service use have been investigated in many epidemiological studies; to date, however, very little research has focused on patients with SMD. Frequent users of psychiatric services (revolving-door patients) have received the most scrutiny [15-17]. While there is no unambiguous definition of 'frequent users' or clear demarcation between 'frequent' and 'infrequent' service users, certain socio-demographic and clinical characteristics associated with patients who make more frequent use of services have come to light [18]. These patients are generally male, young or middle-aged, and live alone. In addition, they lack social support and are often unemployed and homeless. Their main reported diagnoses are schizophrenia and other psychotic disorders, personality disorders, substance abuse, and chronic illness. Non-compliance with medication, numerous previous hospitalisations, and inadequate access to aftercare are also associated with frequent resource use, chiefly frequent readmission to hospital or psychiatric emergency rooms and longer length of in-patient stay, incurring above-average health care costs. Generally, frequent users are treated mainly by psychiatrists [12,19,20]. By comparison, patients with SMD followed by GPs are generally older, female, more educated, and live with a spouse or partner. They also receive more support from their families, have fewer symptoms, are more functional, and use relatively few mental health care services compared with patients treated by psychiatrists [12]. Generally, GPs offer care mainly to patients with mental disorders such as depression and anxiety and request help from psychiatrists and other psychosocial professionals for more complex conditions [21,22].

Some studies have also compared the profile of patients with SMD who use services provided by case managers with those who use more standard services [23]. Case manager patients reportedly receive more assistance, have fewer unmet needs, are more likely to stay in contact with service providers post hospital discharge, and express greater satisfaction with services [24] than patients who are not followed by case managers. Other studies have shown that patients with more frequent contact with case managers have more psychiatric symptoms, worse psychosocial functioning, a poor social network, and reduced quality of life [23,25].

Despite the growing interest in user profiles among patients with SMD and alternatives to hospital-based care, we have found no study that looks at predictors of service-use frequency by patients with SMD involving the three major categories of professionals (psychiatrists, GPs, case managers). No previous study has also compared predictors of service utilisation of various frequent and infrequent users, taking into account concurrent consultation of one or more professionals. Since frequent users account for an estimated 20% to 50% of all psychiatric admissions [26], greater knowledge of their service-use patterns would contribute to efforts to improve service organisation. Given the aims of health care reforms, a clearer understanding of variables that enable or hinder service use by patients with SMD would be of great value.

The health care system in Quebec and Canada is an interesting context in which to study professional service utilisation by patients with SMD. In Canada, health care management is a provincial jurisdiction and has been regionalised over the past two decades. Under the Canada Health Act, all residents are entitled to free in-patient or out-patient care at the point of delivery. Patients receive treatment at publicly funded facilities or are seen by private specialists or GPs in the community who charge their provincial health plan for their services. Almost all psychiatrists practise in hospital settings; most GPs practise in group or solo private clinics. Case managers can work in hospitals or community health centres (known as CLSCs in Quebec). In addition, supervised housing resources and voluntary organisations such as self-help groups are also active health-care providers for patients with SMD. In Quebec, health care and social services are integrated. Several initiatives have been put forward to improve mental health care system integration, including shared care which aims to enhance service coordination among GPs, psychiatrists, and multidisciplinary mental health teams. However, many service providers still work alone, and waiting-time for access to psychiatrist expertise is very long [27,28].

As in most epidemiological studies carried out to predict health care service utilisation, Andersen's behavioural model [29,30] was used to frame our analysis. This model has the merit of encompassing individual and contextual dimensions. It classifies predictors of service use into three categories: predisposing, enabling, and need factors. Predisposing factors are individual characteristics that exist prior to the illness (e.g. socio-demographic profile and attitudes, values, and knowledge about services). Enabling factors refer to various features that influence care delivery and attitudes toward care; they encompass variables such as income, social support, and perception of care satisfaction and adequacy. Need factors include the assessment of physical and mental health both by patients and professionals (e.g. individual's illness or impairment requiring service use).

This study is one of the first to examine predictors of professional consultation for patients with SMD using the Andersen's model. Its aim is to identify and compare variables associated with the use by patients with SMD of services offered by psychiatrists, general practitioners (GP), and case managers. The article also compares variables associated with frequent service use of one professional solely, and groups of two or three professionals respectively, with infrequent or no users (or frequent users of zero professionals). Frequent users are defined in this study as consulting psychiatrist or GP services, respectively, more than once every six months, or case manager services, more than once every two months. We hypothesised that for each type of professional, predictors of patient use frequency would be distinct. We also believe that hindering factors are more likely to be associated with frequent service use as compared with infrequent service use.

## Methods

The study has a cross-sectional design and was conducted in Quebec (Canada). Patients were recruited in five areas in the province, representing urban, semi-urban, and rural areas. Recruitment was carried out from December 2004 to June 2005. Inclusion conditions required that patients: (1) be aged 18 to 65; (2) be living in the community; (3) were hospitalised in the course of the previous year; and (4) were diagnosed with SMD according to ICD-9-CM diagnosis criteria 295 (Schizophrenia), 296 (Episodic Mood disorders, specifically bipolar disorders) or 297 (Delusional disorders). Based on these criteria, patients were selected from a list of 332 individuals whose names were provided by hospital archives in the participating areas. Following verification, 40 patients were excluded because they did not meet all of the above-listed criteria. Another 68 were excluded as their case managers deemed them unfit for participation (extreme severity of disorder, language barriers). In addition, 84 subjects declined the offer to participate. The final sample consisted of 140 subjects out of 224 potential participants, for a response rate of 62.5%.

Data collection was based on patient clinical records and the use of two instruments: the Camberwell Assessment of Needs (CAN) and a questionnaire on service-utilisation patterns. Both instruments were administered by research assistants with a professional clinical background who received special training as part of the study. Patients were interviewed at home or elsewhere at their convenience and were offered modest financial compensation to cover travel expenses and their time. The research was approved by the relevant ethics boards at hospitals and community-based agencies. Each participant was required to sign a consent form after receiving a complete and clear description of the study.

### Measurement instruments and predictors

The CAN is one of the most commonly used instruments for comprehensive needs-assessment in mental health services. Developed by the Health Service Research Department of the Institute of Psychiatry in London, the CAN has been widely studied [31-33]. Its reliability has been demonstrated both for population of different languages and cultures [34-38]. The French version has been validated with long-term hospitalized patients in Quebec, Canada [39]. In addition to assessing patient needs, the CAN may also be used to gauge: help provided by relatives or services; perceived need for help from services; and adequacy of, and satisfaction with, help provided. The CAN questionnaire covers 22 clinical and psychosocial domains, grouped into five needs categories: (i) basic (accommodation, food, daytime activities); (ii) health (physical health, psychotic symptoms, psychological distress, safety to self, safety to others, alcohol use, drug use); (iii) functioning (self-care, looking after the home, child care, basic education, money); (iv) social (company, intimate relationships, sexual expression); and (v) information and utilities (information on the illness and its treatment, transport, telephone, benefits). For each of the 22 domains, patients indicated perceived problems on a three-point scale (no problem = 0, moderate problem = 1, serious problem = 2). If a moderate or serious problem was reported, patients were asked questions on: the level of help received from relatives; the level of help needed and received from services (none = 0, low = 1, moderate = 2, high = 3); and, finally, whether they received the right type (adequate = 1; not adequate = 0) and the right amount of help (satisfied = 1; not satisfied = 0). In every section, patients are permitted to answer, 'I don't know'.

The questionnaire on service utilisation patterns was adapted from the Statistics Canada Canadian Community Health Survey (CCHS 1.2) for patients with SMD [40]. In support of efforts to adapt the questionnaire, a literature review was conducted using Ovid Medline and PubMed research database content from 1996 onward on service-use patterns, care continuity, and shared-care mental health models. The validity of the questionnaire's content was assessed by twelve experts in the field and pre-tested with ten patients not included in the sample. The final version contained 48 items divided into five sections: patterns of service use; consultations with psychiatrists; with GPs; and with case managers, and relationships with family members. Generally, the questionnaire featured open-ended or multiple-choice questions, but also included a few five-point Likert Scale questions.

**Figure 1:**
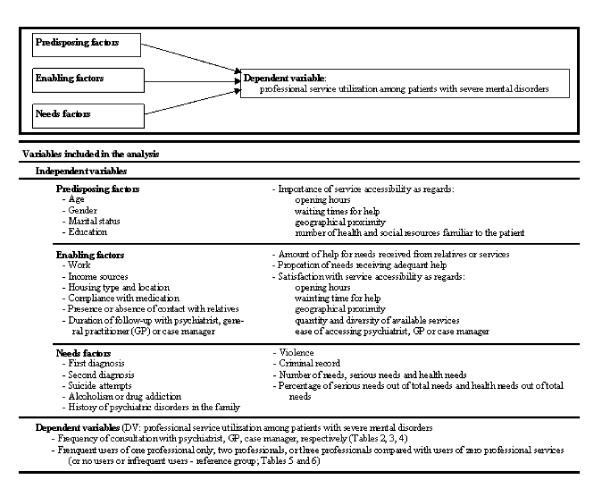
**The Andersen behavioural Model of Health Service Utilisation**.

To gauge predisposing factors, both patient clinical records (age, gender, marital status, and education) and the questionnaire on service utilisation patterns (importance of service accessibility in terms of opening hours, waiting time to getting help, and geographic availability, and number of health and social resources familiar to the patient) were used (Figure 1). Data on potential enabling factors were collected from patient clinical records (work, income sources, housing type and location, compliance with medication) and from the service-use questionnaire (presence or absence of contact with relatives; duration of follow-up with psychiatrist, GP, or case manager; and satisfaction with service accessibility in terms of opening hours, waiting time to access help, and geographic accessibility, quantity and diversity of available services, and ease of accessing a psychiatrist, GP, and case manager). Enabling factors were also gauged using the CAN questionnaire: amount of help for global needs and need categories from relatives or from services, and proportion of needs receiving adequate help. Finally, need factors were extracted from the patient clinical records (first and second diagnoses, suicide attempts, history of psychiatric disorders in the family, alcoholism or drug addiction, violence, and a criminal record) and from the CAN (number of: needs, serious needs, and health needs; and percentage of: serious needs out of total needs and health needs out of total needs).

### Data analyses

All variables included in predisposing, enabling, and need factors were used as independent variables. To identify independent variables associated with the frequency of patient consultation with psychiatrists, GPs, and case managers, respectively, multiple linear regressions were conducted on each of these dependent variables. Frequency of consultation was a continuous variable related to each professional and was measured based on service use in the past 12 months. Patients could answer that they had visited psychiatrists, GPs or case managers zero to several times, on a weekly, monthly or yearly basis. Multinomial logistic regression was used to compare variables associated with infrequent and frequent professional service use. The sample was divided into four groups consisting of frequent professional service users of: all three categories of professionals concurrently; two categories; one category; or none of the categories of professionals (i.e. infrequent professional service users or no users). The line separating frequent from infrequent professional service users was set based on the frequency of consultation per each professional as reported in statistics collected as part of mental health care reforms in Quebec [41]. Frequent users of psychiatrist or GP services were found to consult these professionals, respectively, more than once every six months. Frequent users of case manager services were found to consult these professionals more than once every two months. This cut-off line also represents the median of use-frequency levels (if patients are distributed into equal subcategories).

Both multiple linear and logistic models were preceded by univariate and bivariate analyses. Univariate analysis included verification of normality distribution and calculation of mean values for continuous variables and of frequency distribution for categorical variables. Linear bivariate analyses consisted of testing significant associations among each of the three continuous dependent variables and all independent variables (P ≤ 0.10). All independent variables found, in the preceding step, to be significantly associated were used to build multiple linear regression models using the backward stepwise LR technique (P ≤ 0.05). The final models were assessed as to goodness-of-fit and proportion of variance explained. To build the multinomial logistic regression model, independent variables significantly associated with the four-category dependent variable in bivariate analyses were used (P ≤ 0.10). The improvement of the multiple model as regards goodness-of-fit was achieved using the Likelihood Ratio test: variables yielding Chi-square test with non-significant P value (P > 0.05) were excluded step by step. Adjusted odds ratios (ExpB) were presented that compared frequent users of 1, 2 and 3 professionals with infrequent or non users. Finally, a Nagelkerke Pseudo R-Square was computed to estimate the proportion of variance explained.

## Results

### Description of the sample

Predisposing, enabling, and need factors and service utilisation characteristics are displayed in Table 1. The majority of participants (86 of 140, 61%) were males, with a mean age of 47 years (SD = 12). The sample was compared to non-responding patients for gender distribution and age, which yielded non-significant results (age: t = -1.489; df = 222; P = 0.138); (gender: Pearson χ^2 ^= 0.011; df = 1; P = 1.000). The sample was also compared with all Quebec hospitalised patients for the same year and diagnoses [42], for age (d f = 1611; P = 0.222) and gender distribution (χ^2 ^= 6.331; d f = 1; P = 0.012). No difference between our sample and the Quebec patient population was found with regard to age. A difference was found for gender distribution, with an overrepresentation of men in our sample.

**Table 1:** Predisposing, enabling and need factors and service utilisation characteristics (n = 140)

				Frequency	Percent
**Predisposing factors**		Importance attributed to service accessibility in terms of opening hours	Very important	33	23.6

**Enabling factors**	Amount of help from services for (mean, SD):	functioning needs		2.4	1.2
		information and utility needs		2.0	0.9
		social needs		1.9	0.9
	
	Amount of help from relatives for:	global needs		5.6	3.2
		social needs		1.8	1.09
	
	Duration of follow-up (in years) by (mean, SD):	psychiatrist		3.01	1.42
		general practitioner (GP)		1.78	2.04
		case manager		2.12	1.45
	
	Satisfaction with regard to:	geographic accessibility	Very satisfied	34	24.3
		diversified service accessibility	Very satisfied	28	20.0
		waiting time to receive help	Very satisfied	27	19.3
	
	Satisfaction with ease of accessing a:	GP	Very satisfied	22	15.7
		psychiatrist	Very satisfied	27	19.3
		case manager	Very satisfied	29	20.7
	
**Need factors**	Number of	global needs (mean, SD)		5.9	3.6
		serious needs (mean, SD)		1.9	2.1
		health needs (mean, SD)		2.0	1.4
	
	Diagnoses:	Main diagnosis	Schizoaffective disorders	59	42.1
			Paranoid schizophrenia	49	35.0
			Others schizophrenic disorders	25	17.9
			Delusional disorders	5	3.6
			Bipolar disorders	2	1.4
	
		Second diagnosis	Personality disorders	29	20.7
			Bipolar disorders	5	3.6
			Autism	1	0.7
	
	History of suicide attempts (yes/no)			42	30.0
	Number of suicide attempts (mean, SD)			0.42	0.98
	History of violence (yes/no)			38	27.0
	Psychiatric disorders in the family (yes/no)			63	45.0

**Service utilisation**	Patient distribution according to number of professionals frequently consulted ('frequent users')		0	14	10.0
			1	38	27.1
			2	67	47.9
			3	21	15.0

Most study participants had completed secondary school (n = 106, 76%), and some had a university degree (n = 17, 13%). The vast majority were unmarried (n = 128, 91%). Most lived in urban areas (n = 84, 60%). The predominant source of income was social welfare (n = 94, 70%), though some respondents were employed (n = 15, 11%). As for housing, most participants lived in their own apartment (n = 84, 60%); others in supervised facilities (n = 56, 40%). Service-utilisation distributions revealed a greater use of hospital-based services (n = 122, 87%) than of primary care services (n = 113, 81%). Almost all participants reported being followed by a psychiatrist (n = 130, 93%) and half by a GP (n = 70, 50%). Finally, 84% of participants reported being followed by a case manager (n = 117).

### Modelling frequency of professional service use

Overall, 101 patients with SMD (72%) reported visiting psychiatrists at least once in six months, while the remaining 39 participants (28%) saw them less often or did not consult them. As for GP consultation, 43 patients with SMD (31%) indicated seeing them at least once every 6 months, while the remaining 97 participants (69%) reported visiting them less often or did not consult them. Finally, a total of 91 participants (65%) mentioned visiting case managers at least once per two months, while the remaining 49 (35%) saw them less often or did not use their services. Variables associated with the frequency of psychiatrist, GP, and case manager consultations are displayed, respectively, in Tables 2, 3 and 4. In bivariate analyses, 11 independent variables for psychiatrists, 17 for GPs, and 8 for case managers were significantly associated with the frequency of consultation of these professionals. Variables included in the final regression linear models explain respectively 50%, 61% and 18% of the total variation for psychiatrist, GP, and case manager consultation. In the final model, there are three variables associated with psychiatrist consultation: satisfaction with regard to access to a psychiatrist; access to diversified services; and number of suicide attempts. Four variables associated with GP consultation were identified: satisfaction with regard to access to a GP; duration of patient follow-up by GPs; and amount of help from relatives for social and global needs. Finally, there were two variables associated with case manager consultation: duration of patient follow-up by the case manager; and amount of help from services for information and utility needs.

**Table 2:** Variables associated with the frequency of psychiatrist consultations

	Bivariate analyses Standardized Beta (P value)	Multiple linear regression Standardized Beta (95% CI)
**Enabling factors**		
Housing situation (supervised vs autonomous)	0.157 (0.097)	
Amount of help from services for social needs	0.274 (0.024)	
Duration of follow-up by psychiatrist	0.495 (< 0.001)	
Duration of follow-up by case manager	0.190 (0.043)	
Satisfaction with regard to diversified service accessibility	-0.200 (0.037)	-0.338 (-0.612; 0.184)
Satisfaction as regard waiting time to getting help	0.188 (0.047)	
Satisfaction as regard ease of accessing psychiatrist	0.583 (< 0.001)	0.537 (0.316; 0.630)

**Need factors**		
Schizoaffective disorders as first diagnosis	0.260 (0.005)	
Personality disorders as a second diagnosis	0.326 (< 0.001)	
History of suicide attempts	0.168 (0.075)	
Number of suicide attempts	0.179 (0.058)	0.206 (0.029; 0.483)

		F = 20,935; P < 0,001 R2 = 49,9%

**Table 3:** Variables associated with the frequency of general practitioner (GP) consultations

	Bivariate analyses Standardised Beta (P value)	Multiple linear regression Standardised Beta (95% CI)
**Predisposing factors**		
Age	0.160 (0.068)	
Gender (male = 1)	0.246 (0.005)	
Marital status	0.319 (< 0.001)	
Importance attributed to service accessibility in terms of opening hours	0.295 (0.001)	

**Enabling factors**		
Housing situation (supervised vs autonomous)	-0.173 (0.049)	
Territory (urban vs semi-urban/rural)	-0.263 (0.002)	
Amount of help from relatives for social needs	0.235 (0.033)	0.166 (0.012; 0.622)
Amount of help from relatives for global needs	0.160 (0.068)	0.161 (0.006; 0.183)
Duration of follow-up by GP	0.692 (< 0.001)	0.351 (0.069; 0.470)
Duration of follow-up by case manager	0.261 (0.003)	
Satisfaction with regard to geographic accessibility	0.152 (0.089)	
Satisfaction with regard to waiting time to receive help	0.179 (0.040)	
Satisfaction with regard to ease of accessing a GP	0.738 (< 0.001)	0.425 (0.124; 0.516)

**Need factors**		
Number of health needs	0.178 (0.042)	
History of suicide attempts	0.186 (0.034)	
Number of suicide attempts	0.182 (0.037)	
Psychiatric disorders in the family	0.154 (0.095)	

		F = 26.887; P < 0.001 R2 = 61.3%

**Table 4:** Variables associated with the frequency of case manager consultations

	Bivariate analyses Standardized Beta (P value)	Multiple linear regression Standardized Beta (95% CI)
**Predisposing factors**		
Gender (male = 1)	0.161 (0.058)	
Marital status	0.222 (0.008)	
**Enabling factors**		

Territory (urban vs semi-urban/rural)	-0.206 (0,015)	
Amount of help from services for information and utility needs	0.220 (0.060)	0.186 (0.068; 0.794)
Duration of follow-up by GP	0.142 (0.093)	
Duration of follow-up by case manager	0.441 (< 0.001)	0.356 (0.160; 0.684)
Satisfaction with regard to diversified service accessibility	0.197 (0.022)	
Satisfaction with ease of accessing psychiatrist	0.160 (0.059)	

		F = 7,270; P = 0,001 R2 = 17,6%

### Modelling frequent professional service users as compared with infrequent users

Out of the four user groups, frequent users of two categories of professionals were the largest group, corresponding to 48% of the sample (n = 67 patients). Of this group, 77% (n = 52) were followed by a psychiatrist and case manager, 15% (n = 10) by a GP and case manager, and 8% (n = 5) by a psychiatrist and GP. The second-largest user group consisted of frequent users of only one category of professionals (27%, n = 38), including psychiatrists at 63% (n = 24), case managers at 24% (n = 9), and GPs at 13% (n = 5). Patients reporting high frequency consultation of all three categories of professionals (the third group in importance out of the four user groups) represented 15% (n = 21). Finally, the last group, i.e. users with few consultations or who did not consult professionals, accounted for 10% of the sample (n = 14).

Table 5 presents variables associated with these four user groups in bivariate analyses. The multinomial logistic regression model is displayed in Table 6 (users with a low consultation rate are the control group). The independent variables included in the final model explain 45% of the total variance (Nagelkerke Pseudo R-Square). This model highlights the fact that the duration of patient follow-up by case managers increases the likelihood of more frequent visits with a larger number of professionals. The probability of patients' being frequent users of more categories of professionals also rose with their satisfaction regarding the ease of accessing GPs. Satisfaction regarding the ease of accessing psychiatrists was also found to be significantly associated with frequent users, primarily of one category of professionals only, and secondarily, two categories of professionals.

**Table 5:** Variables associated with the number of frequently used professionals: bivariate multinomial regression (n = 140)

	Frequent users of 1 professional (n = 38)			Frequent users of 2 professionals (n = 67)			Frequent users of 3 professionals (n = 21)		
	**B**	**Sig**.	**Exp(B)**	**B**	**Sig**.	**Exp(B)**	**B**	**Sig**.	**Exp(B)**

**Enabling factors**									
Amount of help from services for functioning needs	0.024	0.941	1.024	-0.194	0.526	0.824	0.63	0.1	1.878
Amount of help from services for information and utility needs	1.837	0.096	6.279	1.889	0.083	6.616	2.445	0.031	11.534
Duration of follow-up by general practitioner (GP)	0.032	0.851	1.032	0.099	0.536	1.104	0.478	0.01	1.612
Duration of follow-up by case manager	0.063	0.789	1.065	0.4	0.072	1.492	0.512	0.047	1.668
Satisfaction with regard to waiting time to receive help	0.045	0.844	1.047	0.277	0.22	1.319	0.671	0.043	1.957
Satisfaction with ease of accessing a GP	-0.038	0.819	0.963	0.106	0.486	1.112	0.725	0.001	2.064
Satisfaction with ease of accessing a psychiatrist	0.308	0.093	1.361	0.466	0.009	1.593	0.536	0.018	1.708

**Need factors**									
History of violence	-1.034	0.123	0.356	-1.044	0.091	0.352	0.192	0.782	1.212

Reference group in the dependent variable: frequent users of zero professionals (n = 14)

**Table 6:** Variables independently associated with the number of frequently used professionals: multiple multinomial regression

	Frequent users of 1 professional (n = 38)			Frequent users of 2 professionals (n = 67)			Frequent users of 3 professionals (n = 21)		
	
	B	**Sig**.	Exp(B)	B	**Sig**.	Exp(B)	B	**Sig**.	Exp(B)
**Enabling factors**									
Amount of help from services for functioning needs	0.059	0.889	1.061	-0.267	0.511	0.765	0.56	0.268	1.75
Duration of follow-up by case manager	0.837	0.056	2.309	0.96	0.025	2.613	1.252	0.01	3.498
Satisfaction with ease of accessing a general practitioner	0.475	0.167	1.608	0.646	0.051	1.908	1.208	0.001	3.346
Satisfaction with ease of accessing a psychiatrist	0.703	0.024	2.02	0.594	0.042	1.811	0.528	0.146	1.696

Nagelkerke Pseudo R-Square: 0.451

Likelihood Ratio Tests: Chi-Square: 45.934; P < 0.001

Reference group in the dependent variable: frequent users of zero professionals (n = 14)

## Discussion

The task of identifying and comparing predictors of service use by category of professionals (psychiatrists, GPs, case managers) contributes to efforts to promote alternatives to hospital-based services [3], which are central to current health care reforms. Very few studies have explored patterns of service use by patients with SMD [43,44]. Our study is one of the first to observe these patients using Andersen's behavioural model. Patients with SMD usually present with characteristics that favour close follow-up by physicians and psychosocial professionals, such as case managers [30]. Since one sample inclusion criterion was that patients must have been hospitalised in the past twelve months, frequent users accounted for the large majority in our sample. Frequent users are a leading priority in current health care reforms as they are highly vulnerable, face major stigmatisation, and generate considerable health care costs [18,26].

Contrary to the literature focusing on the use of Andersen's model in the general population [40], in our regression models, seeking mental health care services from psychiatrists and GPs explained an acceptable level - 50% and 61%, respectively - of the variance. The case manager model was also acceptable, explaining 18% of the variance. In opposition to much of the literature [45-47], of the nine variables associated with service use for the three professional categories in our final regression linear models, only one was related with the need-factor category (i.e. number of suicide attempts). All other predictors were enabling factors. These results are coherent with findings reported by Lemming and Calsyn [30] who also studied utilisation of services for patients with SMD. As the latter are quite a homogenous population - in our sample, almost all had schizophrenia - it is not surprising that the need-factor category is not closely associated with the frequency of professional consultation. Need factors are usually the prime predictors of service use in epidemiological studies involving global populations [45-47].

The only need variable in the multivariate analyses, i.e. number of suicide attempts, was associated with more psychiatrist consultations. Suicide is the leading cause of premature death among patients with schizophrenia [48]. Suicide and suicide attempts occur at a significantly greater rate in persons with schizophrenia than in the general population [49]. It is estimated that 10% to 13% of individuals with schizophrenia commit suicide, and 20% to 40% make suicide attempts [50]. Moreover, recent discharge from hospital (as in our sample) is associated with more suicide and suicide attempts [49,51]. These high-risk patients require greater specialised care, especially from psychiatrists.

Unexpectedly, there was scant difference between variables associated with the frequency of consultation among any of the three professional categories. The eight other predictors of one or another category of professionals involved either the amount of help received (i.e. from relatives for global and social needs or from services for information and utility needs), care access or continuity of care. In the literature [52-54], adequate follow-up of patients with chronic or serious disorders has been found to be closely associated with an extensive range of services received, along with care accessibility and continuity. The positive association between GP consultation and the amount of help received from relatives for global and social needs may signify that patients who consult GPs with greater frequency enjoy a stronger family network. This network may complement services offered by GPs, especially in more intimate areas of a patient's life, involving need domains with which professionals may be less comfortable (such as intimate relationships and sexual expression). A family network may also encourage patients to consult GPs to maintain their level of health [55].

Help provided in the information and utility need category is closely associated with case manager role (who provide information on the illness and its treatment and transport-related issues). The more frequently these professionals are consulted, the greater the amount of help received, especially in this need category [56,57].

In Quebec and Canada, physician care can be difficult to access. It is worth mentioning that the Canadian health care system no longer ranks among the best in the world, due primarily to long waiting times to consult GPs or specialists [1]. An estimated 25% of the population in Quebec is without a GP; therefore, walk-in clinics are the sole health care solution for many people [58]. In our study, satisfaction with ease of access to psychiatrists and GPs was closely associated with more patient consultations. Nevertheless, the greater is the importance given to the frequency of psychiatrist consultation, the lower is patient satisfaction with access to diversified services. Patients seemed either to be 'stuck' with a particular psychiatrist when there was access to them or they consulted a psychiatrist because alternatives were scarce. When there is a shortage of GPs (as is the case currently in Quebec), the likelihood of patients with SMD being transferred to psychiatrists rises since these patients are more challenging and require more frequent care [59]. An alternative hypothesis is that patients may be satisfied with psychiatrist care alone and do not need other types of services. Finally, longitudinal continuity, defined as sustained follow-up over time, is a core component of global care and a major driver of service utilisation for patients with considerable needs, such as individuals with SMD [60-62].

Almost half of the patients in our study were reportedly frequent users of at least two categories of professionals: psychiatrists and case managers. Patients with SMD, especially with schizophrenia and recently hospitalised, usually need specialised care and close professional follow-up for their bio-psycho-social problems [63]. Generally, psychiatrists also recommend that case managers follow patients with major needs [64]. Only a minority of patients (23%) consulted both psychiatrists and GPs. In Quebec, these professionals practice primarily in silo, as shared care (one of the aims of current reforms) is still relatively undeveloped [59]. As a result, GPs are the professionals whom patients with SMD consult least often, probably due to the access problems mentioned above, competing demands from other patients, and insufficient training among GPs in mental health care, and development of shared care [65]. Stigmatisation of patients with SMD by GPs has also been reported as a hindering factor (e.g. GPs' fear of patient crisis and psychotic disorders) [66].

Both service accessibility and longitudinal continuity appear to be core predictors of frequent use of professional health care services, as compared to no or infrequent use in our multinomial regression model. In our findings, the higher were patient satisfaction with access to GPs and to the duration of case manager follow-up, the greater was the number of professionals consulted. This first finding may be due to the shortage of GPs. Recent studies [28,59] have also suggested that GPs tend to transfer patients with mental disorders to specialists, but that collaborative care tends to reverse this trend. In our sample, only 50% of patients had access to GPs who did not necessarily provide continuous care to these patients. The literature on medical homes [67] has emphasised the importance for chronic care patients of having a regular primary care provider, especially a GP involved in care coordination. As for case managers, they generally enable service use and continued contact with service providers following patient discharge from hospital. As for longitudinal continuity, we have already reported its importance as a core component of care quality and driver for service use by patients with SMD [61].

Satisfaction with access to psychiatrists was primarily associated with patients' consulting a single category of professionals and secondarily with two categories of professionals. This finding supports our hypothesis that patients' satisfaction with services provided by psychiatrists reduces their need to consult other providers, such as GPs or case managers. In the great majority of cases where patients were followed by one professional or by two, the professional was or included a psychiatrist. This situation, however, is inconsistent with current trends and is not cost-effective. It also points to the need to raise awareness among the public and health care professionals of the importance of strengthening primary care and shared-care initiatives, which have been shown to provide effective service to patients with chronic disabilities [68]. Finally, as was the case for our multivariate linear analyses, the multinomial model did not corroborate our second hypothesis that hindering factors are more associated with frequent service users as compared with infrequent service users. We believed that a greater number of patient health needs would be associated with a greater volume of GP consultation, but this was not the case.

## Strengths and limitations

The study is of value as it is one of the few that apply Andersen's comprehensive behavioural model to the use of professional health care services by patients with SMD. However, it includes certain limitations that are worth noting. It is a cross-sectional study; a longitudinal study would have allowed us to isolate associations among the different variables more effectively. As our cohort consisted almost exclusively of patients with schizophrenia, we cannot claim that our results are representative of the general population or even of a more diverse population of patients with SMD. Moreover, frequent users constitute the majority in our sample. Identifying predictors of professional consultation for this group is a priority considering the high costs of treating patients with SMD and the stigmatisation that they face. In addition, patients from rural areas or more remote regions (distant from university-affiliated or urban centres where psychiatrists are concentrated) were underrepresented in our sample. Patients who more frequently used psychiatrist services are primarily located in urban settings [69,70]. Compared with the Quebec hospitalised population with SMD, men were also overrepresented in our sample. Finally, the threshold employed to distinguish frequent users from infrequent users was not based on a strong consensus, since the literature provides little in the way of a definition of 'frequent' service users with SMD.

## Conclusion

Contrary to what the literature suggests, our findings are that health care system organisation and professional practice have a greater impact on patient consultation of professional services than do patient need profiles. This finding may be due at least in part to the homogeneity of our study population (primarily frequent users with schizophrenia, recently discharged from hospital). Uncovering system inefficiency with regard to access and care continuity is a major issue in caring for individuals with SMD. GPs prove to be an underutilised resource. As SMD is often associated with comorbidity, such as physical (e.g. diabetes, obesity and hypertension) and substance abuse problems, it is crucial that patients with SMD be followed by GPs. Individuals with SMD should have the same access to care as the general population and receive services that are not stigmatising. Greater effort should be directed at increasing shared-care initiatives, favouring improved coordination among psychiatrists, GPs, and case managers. Generally, good care access, care continuity, and diversified services are strong predictors of quality of care for chronic and seriously disabled patients. While it is true that the province of Quebec provides fairly accessible public health care and is in the midst of bringing about major reforms to enhance integration and primary care, more effort is needed to implement organisational change and reduce stigmatisation for patients with SMD. Such developments would also be welcome in other countries, especially where private health care is more prevalent, as they would raise awareness of the need for adequate care access and care continuity for persons with serious and disabling disorders.

## Abbreviations

CAN: Camberwell Assessment of Need; CLSC: Community Local Service centre; GP: General practitioner; SMD: Severe mental disorders.

## Competing interests

The authors declare that they have no competing interests.

## Authors' contributions

MJF designed the study. JMB made the statistic analyses with the help of JC. MJF and GG wrote the article. All authors have read and approved the final manuscript.

## Pre-publication history

The pre-publication history for this paper can be accessed here:

http://www.biomedcentral.com/1472-6963/10/141/prepub
